# Anesthetic management of conjoined twins undergoing one-stage surgical separation: A single center experience

**DOI:** 10.12669/pjms.292.3275

**Published:** 2013-04

**Authors:** He-Jiang Zhong, Hong Li, Zhi-Yong Du, He Huan, Tian-De Yang, Yue-Yong Qi

**Affiliations:** 1 He-Jiang Zhong, MD, Department of Anesthesiology, Xinqiao Hospital, Third Military Medical University, Chongqing 400037, China.; 2 Hong Li, MD, Department of Anesthesiology, Xinqiao Hospital, Third Military Medical University, Chongqing 400037, China.; 3 Zhi-Yong Du, MD, Department of Anesthesiology, Xinqiao Hospital, Third Military Medical University, Chongqing 400037, China.; 4 He Huan, MD, Department of Anesthesiology, Xinqiao Hospital, Third Military Medical University, Chongqing 400037, China.; 5 Tian-De Yang, MD, Department of Anesthesiology, Xinqiao Hospital, Third Military Medical University, Chongqing 400037, China.; 6 Yue-Yong Qi, MD, Department of Radiology, Xinqiao Hospital, Third Military Medical University, Chongqing 400037, China.

**Keywords:** Conjoined twins, Separation, Anesthetic management, Surgery

## Abstract

***Objective:*** To summarize our experience in the anesthetic management of conjoined twins undergoing one-stage surgical separation.

***Methodology:*** Medical records of conjoined twins admitted to our hospital for treatment and considered for surgical separation from 1996 to present were retrospectively reviewed. Four cases of conjoined twins underwent one-stage surgical separation under general anesthesia. Preoperative evaluation was performed to determine the extent of anatomical conjunction and associated anomalies. Anesthesia was simultaneously induced in all conjoined twins. The intubation procedure was successfully performed with the head slightly rotated to each baby’s side, followed by the administration of vecuronium. Anesthetic agents were administered according to the estimated weight of each baby. One case of conjoined twins underwent surgical separation with cardiopulmonary bypass due to shared hearts.

***Results***: All conjoined twins were successfully separated. No significant respiratory or cardiac events occurred during surgery except for one twin, which died after separation because of complicated congenital heart disease.

***Conclusions*** *: *Accurate preoperative evaluation, respiratory and circulatory management, and close cooperation of the multidisciplinary team are important aspects of anesthetic management of conjoined twins surgery.

## INTRODUCTION

Conjoined twins represent an uncommon congenital malformation, with an estimated incidence of 1: 50,000–1: 100,000,^1^ and approximate 75% of cases are females.^2^^,^^3^ Usually, conjoined twins are classified according to the most prominent site of conjunction: thorax (thoracopagus) 40%, abdomen (xiphopagus and omphalopagus) 33%, sacrum (pygopagus) 18%, pelvis (ischiopagus) 6%, and craniopagus (1-2%).^3^^,^^4^

Conjoined twins usually present complex anatomical conjunction, multiple congenital anomalies, and different extents of cross-circulation. These pose multiple challenges in anesthetic management, particularly in per-operative evaluation, anesthesia induction, airway management, and maintenance of hemodynamic stability. In 1996, the first case of conjoined twins was admitted to our hospital, and since then, four cases have been undergone surgical separation.

The purpose of this case series was to summarize our experience in the anesthetic management of these conjoined twins who underwent one-stage surgical separation, and to review our anesthetic management strategies.

## METHODOLOGY

Approval for analysis and publication of these data was obtained from the Institutional Review Board of the Xinqiao Hospital of Third Military Medical University (Chongqing, China). From 1996 to present, all conjoined twins admitted to our hospital and underwent surgical separation were retrospectively reviewed, and informed consent was given by parents or guardians. Careful physical examination and laboratory tests were performed in all conjoined twins. A detailed treatment plan was designed, according to the conditions of each case of twins.

Four cases of conjoined twins had undergone surgical separation. Anesthesia team, anesthesia machine, and monitoring system were duplicated. Anesthesia was simultaneously induced for each case of conjoined twins. Anesthetic agents were calculated according to the estimated weight of each baby and administered individually. The first case of twins was sedated with ketamine and gamma-hydroxybutyrate. The separation procedure was performed under local infiltration of 0.25% procaine. In cases 2, 3, and 4, babies were induced with ketamine, midazolam, and fentanyl. All twins were facing each other, which hindered the intubation procedure. The heads of both babies were rotated, allowing a reasonable intubating position. When one baby was nasotracheally intubated with the Mcgill’s forceps, the other one was continuously preoxygenated via a face mask with hand ventilation. As soon as the correct position of the tube was ensured, the procedure was repeated on the other twin. All babies were intubated without difficulty after administration of vecuronium. The lungs were ventilated using pressure-controlled ventilation with 70–80% oxygen. Anesthesia was maintained with ketamine, propofol, isoflurane or sevoflurane, fentanyl, and repeated doses of vecuronium, as required.

One-stage surgical separation was performed through an incision made on the skin bridge between twins in each case. In case 4, cardiac separation and reconstruction of twin B’s heart were performed under cardiopulmonary bypass (CPB). At the end of the surgery, all babies were transferred to the ICU for close observation and care. Long term follow-up was performed after hospital discharge.

## RESULTS

***Patient characteristics: ***From 1996 to present, four cases of conjoined twins were admitted to our hospital and had undergone surgical separation. These included three cases of thoracopagus and one xipho-omphalopagus. All conjoined twins’ characteristics are shown in Table-I. Three of the four cases were male, the birth weight ranged between 4.86 and 5.7 kg, averaging 5.315 kg.

All conjoined twins shared livers, and case 4 shared hearts. Other shared organs included sternum (cases 2, 3, and 4) and pericardium (cases 2 and 4). In case 4, inferior vena cava (IVC) and cholecyst were shared. Cardiovascular anomalies were detected in all conjoined twins, particularly in cases 1 and 4. Twin A of case 1 had corrected transposition of the great arteries (CTGA) and ventricular septal defect (VSD). In case 4, twin A presented with a single ventricle and complicated with aortectasis. Twin B also had congenital heart defects.

***Intraoperative anesthetic management: ***Four cases of conjoined twins had undergone surgical separation under general anesthesia. Table-II provides a summary of anesthetic management during surgery. The weight before separation was 4.45–9.95 kg, averaging 7.184 kg.

In case 1, babies were kept on spontaneous breathing during surgery, except when abdominal closure required muscular relaxation. The hand ventilation was used after administration of succinylcholine in each baby. The intraoperative period was uneventful. These were also confirmed by the clinical observation of good chest expansion, satisfactory skin color, and stable vital signs.

In cases 2, 3, and 4, adequate oxygenation, ventilation and operative conditions were achieved during surgery. Because babies required high respiratory rates during separation of shared livers, it was necessary to hand-ventilate each baby during this procedure. In case 4, the separation procedure was performed under CPB due to shared hearts. Twin A presented with complex congenital heart disease, which unfortunately lead to death after separation. Twin B was extubated at the end of surgery and reintubated nasally with a wire-reinforced tracheal tube to simplify postoperative ventilatory support.

During surgery, the total estimated blood loss ranged between 50 and 300 ml, averaging 106 ml. Blood units were administered according to hemoglobin level (targeted level > 10 g/dl). The amount of blood products administered varied between 80 and 450 ml. Warming blankets were placed under and on top of the babies, and all intravenous and irrigation fluids were prewarmed. The mean rectal temperature was maintained at 36 ± 1.6 ^o^C. The mean surgical time was 214 minutes, and mean anesthesia time was 311 minutes. In case 4, the CPB time was 105 minutes. No significant respiratory or cardiac events occurred in these babies during surgery.

***Postoperative care and outcomes: ***The postoperative care and outcomes are shown in Table-III. Twin A of case one developed aspiration pneumonia on postoperative day 3 and died of pneumonia at 36 days after separation. In cases 2 and 3, the postoperative period was uneventful. However, twin B of case 2 died of pneumonia at 5 years of age. Each child of case 3 had returned to our hospital for correction of thoracic deformity (CTD) 4 years after their separation. As for twin B of case 4, cardiac arrest occurred at 10 h after surgery. Open-chest cardiac massage was performed in the ICU. Minutes later the baby regained a normal heartbeat. However, the sternum could not be closed without producing hypotension. Delayed sternal closure (DSC) was performed on postoperative day 5. Unfortunately, this baby died of disseminated intravascular coagulation (DIC) and multiple organ dysfunction failure (MODF) at 29 days after separation. All survivors (n=4) in this study are now living healthily at home except for twin A of case 3, which presents epilepsy.

**Table-I:** Clinical characteristics of conjoined twins

*Case*	*Year*	*Type*	*Sex*	*Birth weight (kg)*	*Shared organs*	*Associated anomalies*	*Age at operation* *(days)*	*Treatment*
1	1996	XO	M	5.5	Liver	A: CTGA, VSD	28	Elective separation
						B: None		
2	2000	TO	M	5.7	Sternum, pericardium, liver	A: ASD	96	Elective separation
						B: PDA		
3	2006	TO	M	5.2	Sternum, liver	A: None	91	Elective separation
						B: PFO		
4	2010	TO	F	4.86	Sternum, pericardium, heart, inferior vena cava, liver, cholecyst	A: Single ventricle, aortectasis	24	Emergent separation
						B: Congenital heart defects^#^		

XO = Xipho-omphalopagus; TO = Thoracopagus; M = Male; F = Female; CTGA = Corrected transposition of the great arteries; VSD = Ventricular septal defect; PDA = Patent ductus arteriosus; PFO = Patent foramen ovale. ^#^Detailed cardiac defects was not available

**Table-II:** Intraoperative anesthetic management

*Case*		*Weight before separation (kg)*	*Loss of blood (ml)*	*Blood products (ml)*	*Fluids (ml)*	*CPB time (min)*	*Time of surgery (min)*	*Time of anesthesia (min)*
1	A	4.895	60	WB: 80	70	-	152	270
	B		65	WB: 80	90	-	157	270
2	A	9.4	140	WB: 260	325	-	285	385
	B		50	WB: 150	310	-	270	385
3	A	9.95	50	PRBC: 100 FFP: 50	270	-	180	220
	B		75	PRBC: 100 FFP: 50	305	-	185	220
4	A	4.45	-	-	-	-	115	235
	B		300	PRBC: 350 FFP: 100	874.5	105	370	500

WB = Whole blood; PRBC = Packed red blood cells; FFP = Fresh frozen plasma

**Table-III:** Postoperative care and outcomes

*Case*		*Extubation time after operation (days)*	*Time in ICU (days)*	*Stay at hospital (days)*	*Complications*	*Surgery after separation*	*Outcome*
1	A	-	36	60	Aspiration pneumonia	-	Died at 36 days after separation
	B	-	17	45	-	-	Alive
2	A	3	5	74	-	-	Alive
	B	3	5	74	-	-	Died at 5 years after separation
3	A	4	5	146	Thoracic deformity	CTD	Alive, epilepsy
	B	4	5	146	Thoracic deformity	CTD	Alive
4	A	-	-	4	-	-	Died at separation
	B	-	29	32	MODF, DIC	DSC	Died at 29 days after separation

ICU = Intensive care unit; MODF = Multiple organ dysfunction failure; DIC = Disseminated intravascular coagulation; CTD = Correct thoracic deformity; DSC = Delayed sternal closure

## DISCUSSION

This study summarizes the anesthetic management of conjoined twins who were admitted to our hospital for surgical separation. Detailed preoperative evaluation, careful intraoperative anesthetic management, and postoperative care are crucial to ensure a successful separation and a safe perioperative course.

Accurate evaluation of shared organs and associated anomalies is crucial for anesthetic management. It is necessary to define the anatomy and vascular supply of the shared organs, especially for the vital organs, such as the heart, liver, and gastrointestinal system. The successful separation of thoracopagus twins is heavily dependent on the degree of cardiac fusion.^5^ Gastrointestinal contrast examination may help to detect the conjunction of the gastrointestinal tract. MRI angiography combined with three-dimensional imaging may provide more information about shared organs.^6^

Although elective separation is probably preferred, emergent separation must be performed when the condition of conjoined twins deteriorates to a life threatening degree, such as one twin being difficult to resuscitate or being stillborn, severe injury of the connecting bridge during delivery, severely compromised hemodynamics or respiration, and correctable life-threatening congenital anomaly.^2^^,^^6^^-^^8^ However, emergent separation has a markedly higher mortality rate (70%–80%) than elective separation.^2^ In case 4, twin A deteriorated progressively towards respiratory failure with severe metabolic acidosis, the babies requiring emergent separation. Unfortunately, they presented with complex cardiac fusion, and twin A died after separation. The other baby died of MODF 29 days after separation.

General anesthesia with tracheal intubation might be preferable in conjoined twins undergoing surgical separation.^4^^,^^9^^-^^10^ Caudal epidural anesthesia combined with general anesthesia was also described in omphalopagus twins.^11^ Surgical separation of case one was performed under local anesthesia plus general anesthesia without tracheal intubation. Spontaneous breathing with 100% oxygen by face mask, careful attention to drug usage, and cautious respiratory monitoring were important factors for maintaining adequate oxygenation and ventilation during surgery. In spite of successful separation, we believe these anesthetic procedures should only be considered for twins with a simple conjunction. Tracheal intubation with mechanical ventilation would have more advantages in airway management during surgery.

Anesthetic induction may be very challenging and fraught with potential risks in conjoined twins. Induction agents and muscle relaxants administered to one baby can cause airway obstruction, hypoventilation, or apnea in the other one, particularly in conjoined twins with marked cross-circulation. On the basis of these concerns, anesthesia was simultaneously induced in our conjoined twins. Furthermore, anesthetic induction should start only when both babies can be mask-ventilated. Induction agents should be administered in relation with the weight of each baby individually.^11^ However, the exact weight of each baby is not available in conjoined twins. The approximate weight may be estimated according to the relative size. Meanwhile, in order to reduce adverse effects of anesthetics, all intravenous agents were carefully administered in reduced incremental doses.^4^^,^^10^

Tracheal intubation can be difficult in conjoined twins due to abnormal anatomy positioning.^12^ Muscle relaxant should be administered when the airway is reliably secured in both babies.^8^ Some authors have described that tracheal intubation was performed in conjoined twins while holding one baby above the other.^5^ However, other authors insisted this procedure would cause auto-transfusion between twins and induce hemodynamic disturbances,^8^^,^^9^ particularly in conjoined twins with significant cross-circulation. In our study, intubation procedures were successfully performed with the head slightly rotated to each baby’s side, following administration of vecuronium. During this procedure, it was necessary to require another anesthesiologist to perform mask-assisted ventilation in the other baby. Some authors have suggested an awake tracheal intubation should be recommended to prevent pulmonary aspiration and airway obstruction, as well as avoiding suspected difficult intubation and significant cross-circulation between twins. However, an awake tracheal intubation may generate several problems, including coughing and straining, which can increase the risk of laryngospasm and induce serious cardiovascular responses.^4^^,^^11^

It is also critical to maintain the circulating volume and hemodynamic stability during surgery. We replaced fluid and blood products on the basis of arterial pressure, CVP, peripheral perfusion and hematocrit. However, because of some extent of cross-circulation between twins, it is difficult to precisely determine blood loss from each baby. Usually, weighing the gauzes, measuring the volumes in the vacuum suction bottles and serial hematocrits, contributed to determining the blood loss. The two anesthetic teams should be in close communication regarding blood loss evaluation for each baby.

Postoperative care, including mechanical ventilation, regulation of fluid and electrolyte balance, prevention of infection, and maintenance of hemodynamic stability, should be carefully managed.^2^ For simple procedures, postoperative ventilation may not be necessary. For prolonged surgery, postoperative mechanical ventilation may be required.

## CONCLUSION

Meticulous anesthetic management is a central factor during surgical separation. Accurate preoperative evaluation, careful monitoring, maintenance of adequate ventilation, and, most of all, close cooperation between all members of the multidisciplinary team, guarantee a successful anesthetic management.

## Authors Contributions

He-Jiang Zhong, Hong Li, Zhi-Yong Du, He Huan, and Tian-De Yang provided perioperative care to the patients. He-Jiang Zhong was involved in acquisition of data and wrote the original draft of the manuscript. Yue-Yong Qi was involved in acquisition of data. Tian-De Yang was involved in the critical revision of the intellectual content of the manuscript.
